# Description of the larva of *Mitosynum vockerothi* Campbell, 1982, with remarks on the adult male genital morphology (Coleoptera, Staphylinidae, Oxytelinae)

**DOI:** 10.3897/zookeys.573.7972

**Published:** 2016-03-24

**Authors:** György Makranczy, Reginald P. Webster

**Affiliations:** 1 Department of Zoology, Hungarian Natural History Museum, H-1088 Budapest, Baross utca 13, Hungary; 2 24 Mill Stream Drive, Charters Settlement, NB, Canada E3C 1X1

**Keywords:** Staphylinidae, Oxytelinae, Mitosynum vockerothi, male genital characters, New Brunswick, Canada

## Abstract

The previously unknown larva of *Mitosynum vockerothi* Campbell, 1982, is described and illustrated. Adult male terminalia and genitalia are illustrated with line drawings. Adults of this species exhibit little difference in size or external morphology between males and females.

## Introduction

The genus *Mitosynum* Campbell, 1982, includes a single species that is endemic to New Brunswick, Canada (Herman 2001, Webster et al. 2012). The description by Campbell (1982) was based on two female specimens, with the habitus illustrated by a line drawing, but genital morphology was not discussed, no measurements of the adults were provided, and the larva was unknown. The first color habitus photo appeared in Makranczy (2006). A subsequent contribution included new records for the genus and species, plus a color image of the adult and the first black and white photographic images of the aedeagus (Webster et al. 2012). The genus is now included in the tribe Syntomiini Böving & Craighead, 1931 (Khachikov 2012). The former Deleasterini Reitter, 1909 included six genera (*Platydeleaster* Schülke, 2003, *Deleaster* Erichson, 1839, *Syntomium* Curtis, 1828, *Mitosynum* Campbell, 1982, *Euphanias* Fairmaire & Laboulbène, 1856 and *Oxypius* Newton, 1982) and was treated as the Euphaniini Reitter, 1909 by Bouchard et al. (2011), the latter name originally proposed at a higher rank and therefore being of priority. This assemblage of genera is heterogenous, as it includes three monobasic genera and their morphological diversities are poorly mapped, and none were ever revised. Khachikov (2012) speculatively modified the tribal classification, based on a few dissections made on common Palaearctic representatives. This involved breaking up the aforementioned assemblage into three different tribes. This system is at best considered tentative and more analyses are required based on additional characters to test the new hypothesis, with the inclusion of the exotic taxa and assessing the variabilities of the wider distributed, non-monobasic genera. Within the former group of six genera, the larvae of *Deleaster* Erichson, 1839 and *Syntomium* Curtis, 1828 are known, but the only complete description is provided for *Oxypius* Newton, 1982, an Austral endemic relict (Newton 1982). In this contribution, we provide a description of the previously unknown larva of *Mitosynum* and male genitalia that will be useful for a future more detailed analysis of the above tribal classification. In addition, we provide measurements of various structures of the adult not included in the original description.

## Material and methods

One larval specimen of *Mitosynum vocherothi*, not mentioned by Webster et al. (2012) was collected along with the nine adults reported in that paper. No other Oxytelinae were found at the site and habitat where the larva was found, a large hummock of *Sphagnum* and *Polytrichum commune* Hedw. near a pond margin (Webster et al. 2012). The specimen bears the characteristic metallic luster of the adults (Fig. 6; see Figure 1 in Webster et al. 2012 for comparison with the adult), leaving little doubt about the correct assignment of the larval specimen. It is unlikely a mature larva, as the size is significantly smaller than that of the adults. However, due to the rarity of this species, we describe the larva here. Based on two male, two female, and four unsexed adult specimens, we also describe and discuss some genitalia features of the adult male and provide measurements of some key structures not included in the original description by Campbell (1982).

**Figures 1–7.**
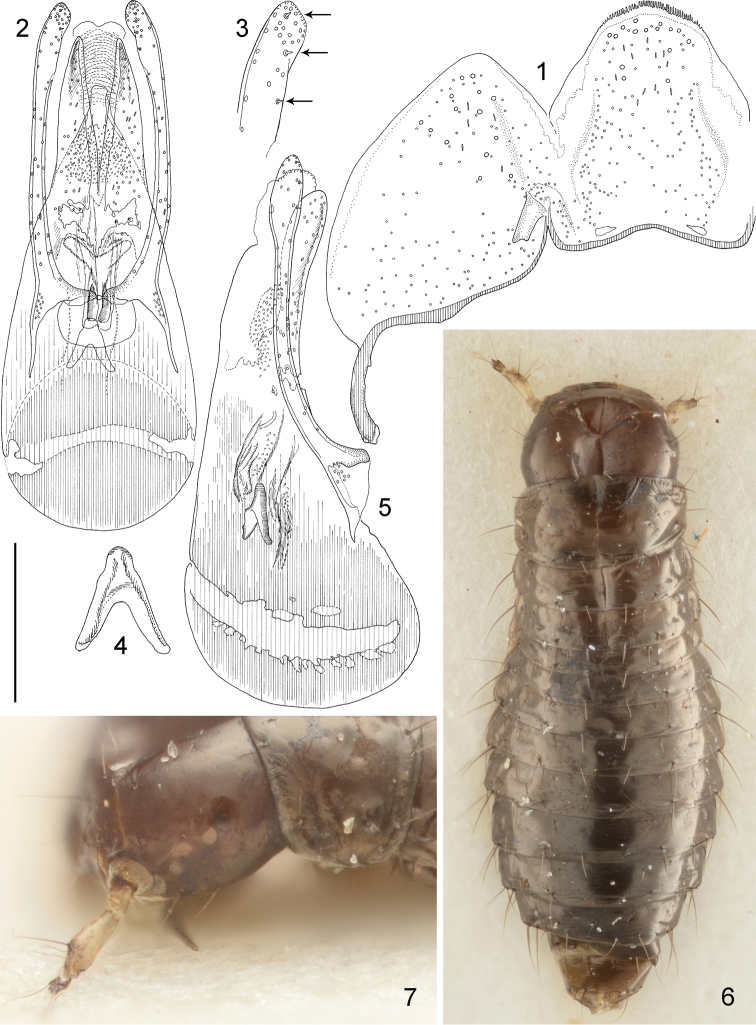
*Mitosynum vockerothi* Campbell, 1982 adult male (**1–5**) and larva (L_2_?) (**6–7**). **1** tergites IX and X **2** aedeagus, “frontal” view **3** apex of paramere **4** sclerite of internal sac **5** aedeagus, lateral view **6** habitus (dry mounted) **7** side of head. Scale bar: 0.1 mm (**3-4**), 0.2 mm (**2, 5**), 0.25 mm (**1**), 0.3 mm (**7**), 0.6 mm (**6**).

The larval specimen was originally dry mounted but subsequently cleared and examined in glycerol following a protocol established in Makranczy (2016). Adult genitalia drawings were made after embedding them into Euparal mounting medium on small plastic slides that were pinned with the specimens according to Makranczy (2006). As the aedeagus is very similar to that in *Deleaster* Erichson, 1839, the illustration technique used by Cuccodoro and Makranczy (2013) was applied. Drawing was done with a Jenalab (Carl Zeiss, Jena) compound microscope and drawing tube (camera lucida). For color habitus photography, a Nikon D4 camera with Mitutoyo PlanApo 5x ELDW lens was used, and layers montaged with ZereneStacker.

The examined specimens are deposited in the Canadian National Collection of Insects, Arachnids, and Nematodes, Ottawa, Canada (CNC), the Hungarian Natural History Museum, Budapest, Hungary (HNHM), and the private collection of Reginald Webster, Charters Settlement, New Brunswick, Canada (RWC).

## Results

### Mitosynum vockerothi

Taxon classificationAnimaliaColeopteraStaphylinidae

Campbell, 1982

Figs 1–7 , 8–14 , 15–20

Mitosynum vockerothi Campbell, 1982: 690.

#### Material examined.

CANADA, New Brunswick, Charlotte Co., near New River, 45.21176°N, 66.61790°W, 7.V.2007, R. P. Webster, small pond & marsh, sifting sphagnum and *Polytrichum commune* on hummock near margin of pond (1 ♂, HNHM; 1 ♂, 1 ♀, 2 sex undetermined, RWC), same data but 7.VII.2006 (1 ♀, HNHM; 1 sex undetermined, RWC; 1 larva L_2_?, CNC), same data but 16.VII.2010 (1 sex undetermined, RWC).

#### Partial redescription of adult morphology.

Measurements in mm (n = 8; 2 ♂, 2 ♀, 4 sex undetermined) showing size range with mean in parentheses: head width at eyes = 0.77–0.84 (0.80); head width at temples = 0.78–0.88 (0.82); maximum width of pronotum = 0.87–0.96 (0.92); approximate width of humeri = 0.84–0.92 (0.88); maximum width of abdomen = 1.09–1.21 (1.14); head length at the midline from front margin of clypeus to the beginning of neck = 0.55–0.60 (0.58); eye length = 0.25–0.29 (0.27); length of temple = 0.14–0.17 (0.16); length of pronotum at the midline = 0.67–0.74 (0.70); length of elytra from shoulder = 0.64–0.73 (0.69); length of elytra from hind apex of scutellum = 0.58–0.67 (0.61); forebody length = 1.92–2.12 (2.02); approximate body length = 3.76–4.35 (4.06). All measured from dorsal view.

#### Male terminalia and genitalia.

Sternite VIII apex in males almost truncate but with rounded corners. Tergite IX with strongly developed but short ventral strut. Tergite X strongly attached (Fig. 1). Aedeagus (Figs 2–5) very strongly sclerotized, dark. Apical opening processes of median lobe strong and elongate. Parameres elongate and rounded at apices. Apical part of paramere (Fig. 3) with a couple of very short peg-like setae. Internal sac with a sclerotized, symmetrical sclerite (Figs 4, 5).

#### Comments.

The sample size was inadequate for a statistical comparison of the size between males and females. However, there was little variation in size among individuals in any of the characters that were measured, indicating sexual dimorphism in size in this species is minimal. Sternite VIII apex in female broadly rounded but medially a little more narrowly than in males, otherwise there are no differences in external morphology between males and females.

The apical opening processes of the aedeagus and parameres very strongly resemble those in *Deleaster* Erichson, 1939. The presence of a few very short peg-like setae (Fig. 3) on the apical part of paramere was not previously known in Oxytelinae. The distinct, symmetrical inner sclerite is the first observation of this character within the six genera of Deleasterini sensu Makranczy (2006).

#### The larva of *Mitosynum vockerothi*

**Figures 8–14.**
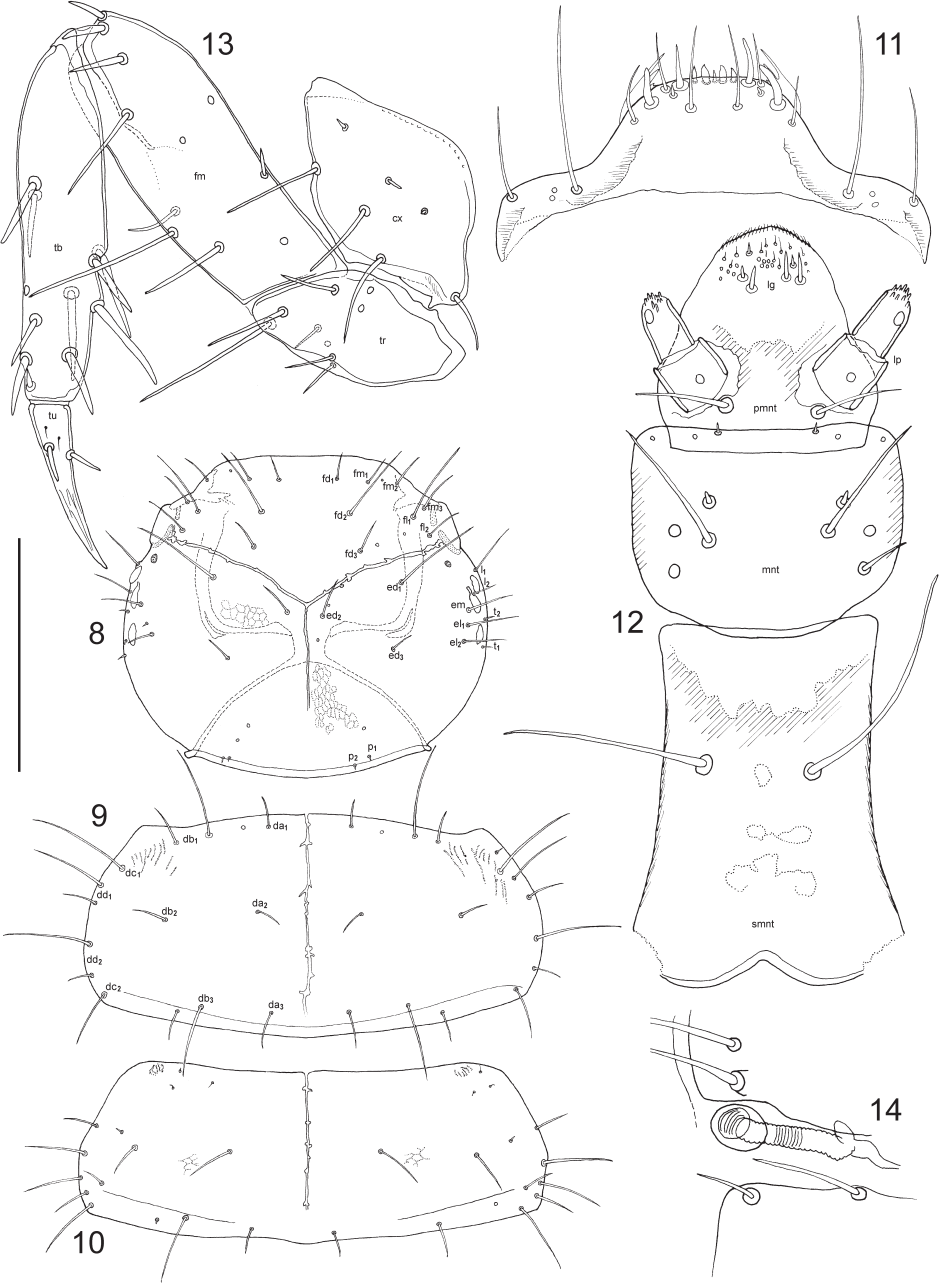
*Mitosynum vockerothi* Campbell, 1982 larva (L_2_?). **8** head **9** pronotum **10** mesonotum **11** labrum **12** labium **13** anterior leg **14** lateral view of spiracle at 1^st^ tergite. Scale bar: 0.1 mm (**12, 14**), 0.17mm (**11, 13**), 0,4 mm (**8–10**).

**Figures 15–20.**
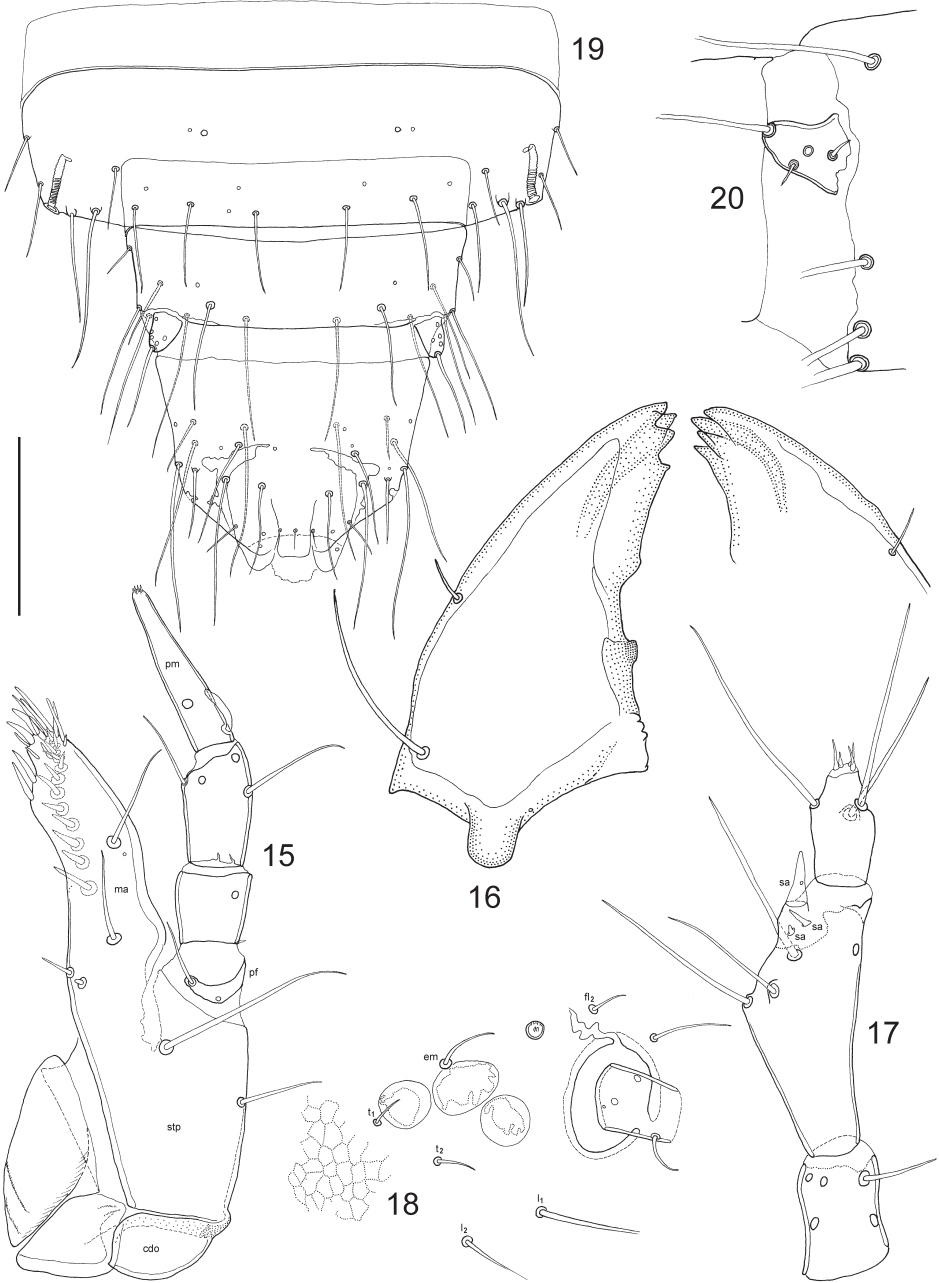
*Mitosynum vockerothi* Campbell, 1982 larva (L_2_?). **15** maxilla **16** mandible **17** antenna **18** ocelli and stemmata, lateral view **19** abdominal segments VIII-X **20** urogomphus, lateral view. Scale bar: 0.1 mm (**16-17, 20**), 0.11 mm (**15**), 0.14 mm (**18**), 0.22 mm (**19**).

(instar unknown but presumed L_2_). Length (in mm): 3.00 [epicranium width (ew) = 0.61]. Body (Fig. 6) at places (e.g. dorsum of head) covered with isodiametric microsculpture, but appearing shiny with metallic luster. ***Head*.** Head capsule (Fig. 8) dorso-ventrally flattened, rounded but slightly transverse, supraantennal prominences elongate. Three stemmata in a strongly curved line (Fig. 18) appearing as lighter bumps on the sideline (Fig. 7). Ecdysial lines end in antennal foramen and a ‘glandular area’ (Newton 1982) is observed posterior to the latter. Dorsum of head with frontal setae (frontal dorsal = fd, frontal lateral = fl, frontal marginal = fm), epicranial setae (epicranial dorsal = ed, epicranial lateral = el, epicranial marginal = em), temporal (t), lateral (l) and posterior setae (p). Antenna (Fig. 17) three segmented, three sensory appendages (sa) on penultimate article, four solenidia (so) on apical. Labrum (Fig. 11) medially trapeziform, laterally transversely elongate, frontal margin with two pairs of stout setae. (Note: The labrum in the examined larva appears to be teratological; only the healthy part is considered and is mostly mirrored for the drawing.) Mandible (Fig. 16) triangular shaped but thick at base, gradually narrowing toward quadrifurcate apex; apical teeth in close proximity. Maxilla (Fig. 15) with cardo (cdo) subtriangular, stipes (stp) apically slightly broadening, with mala (ma) forming an almost uniform plate, palpifer (pf) distinct, maxillary palp three segmented, third palpomere (pm) with digitiform sensory appendage at base. Labium (Fig. 12) with mentum (mnt) subrectangular, slightly transverse, posterior corners rounded, ligula (lg) fused with prementum (pmnt), submentum (smnt) quite elongate, parallel sided, labial palp (lp) two segmented. ***Thorax*.** Tergites with short and long setae arranged more or less along transversal lines; pronotum as in Fig. 9, mesonotum as in Fig. 10, metanotum with setation identical to that of mesonotum. Pronotal discal setae in rows (a-d). Legs with five articles, coxa (cx) rhomboid, trochanter (tr) with a few campaniform sensilla on both sides, femur (fm) with a few scattered pores, tibia (tb) with one pore, apically one tiny spine, tarsungulus (tu) with two small setae in basal position, one on each side. Anterior leg as in Fig. 13. ***Abdomen*.** Segments I-VIII composed of tergites and sternites. First tergum with spiracles at its sides (Fig. 14) in the intersegmental membrane, segments II–VIII with spiracles in tergites (Fig. 19). Abdominal segments IX and X (Fig. 19) with dorsal and ventral sclerites fused. Urogomphi (Figs 19–20) one articled, very short, almost vestigial. Anal lobes not everted in examined specimen but without conspicuous structures.

## Supplementary Material

XML Treatment for Mitosynum vockerothi
